# Peripartum management of a patient with catecholaminergic polymorphic ventricular tachycardia

**DOI:** 10.1111/anec.12796

**Published:** 2020-09-26

**Authors:** Erick Jimenez, Daniel Cortez, Mark McGill, Matthew Ambrose

**Affiliations:** ^1^ Division of Pediatric Cardiology Department of Pediatrics University of Minnesota Minneapolis Minnesota USA

**Keywords:** arrhythmia, beta‐blockers, palpitations, pregnancy, ventricular tachycardia

## Abstract

Catecholaminergic polymorphic ventricular tachycardia (CPVT) is a potentially lethal cardiac channelopathy characterized by episodes of ventricular tachycardia (VT) during exercise or in stressful situations. As the peripartum period creates a stressful environment, we describe our approach of this rare condition in a very common situation, child birth.

## 1 CASE REPORT

A 28‐year‐old female, with her third pregnancy (one completed to term and one spontaneous abortion with unknown cause) at 39 weeks of gestational age and with a history of CPVT type 1 (pathological mutation c.506G > A in the ryanodine receptor [RYR2] gene) (Hsueh, Weng, & Chen, 2006), underwent secondary prevention single‐chamber, single‐coil Boston Scientific Energen implantable cardioverter defibrillator (ICD) placement after she had a sudden cardiac arrest at 23 years of age. The cardiac arrest occurred while the patient was driving, and she was found to be in ventricular tachyarrhythmia by the police. At that point, etiology of the arrest had not been determined and genetic testing had not been performed due to cost limitations.

Of note, the patient's first pregnancy and vaginal delivery occurred prior to her episode of sudden cardiac arrest. These events were uncomplicated and resulted in a child who had sudden cardiac death at 2 years of age (previously asymptomatic); autopsy did not show any abnormalities and DNA testing diagnosed CPVT due to a RyR2 mutation. This eventually led to the genetic evaluation and diagnosis in our patient.

Our patient presented at 23 weeks of gestational age for a fetal cardiology evaluation. She was asymptomatic, except for occasional lower extremities edema. Her cardiovascular examination was normal. She was taking metoprolol 25 mg daily and flecainide 50 mg twice daily before, and it was continued throughout the entire pregnancy. Her ICD was programmed with VVI pacing at 35 bpm, with a ventricular tachycardia (VT) monitor zone at 190 bpm and only one therapy zone of a ventricular fibrillation (VF) zone at 240bpm (anti‐tachycardia pacing [ATP] only during charge, then shock). Her fetal echocardiography was normal. During follow‐up, interrogation of ICD showed no history of ATP or shock therapy delivered. It also demonstrated 3 episodes of non‐sustained VT (NSVT) (9–18 s at rates of 217–254 bpm) in the first trimester. Episodes of NSVT became more frequent (about once a week from 29 to 33 WGA) and she remained asymptomatic during these events. ICD settings and medications doses were kept unchanged throughout the pregnancy. Given the episodes of NSVT, the metoprolol was increased to 25 mg twice daily.

A multidisciplinary meeting, attended by maternal fetal medicine, cardiology (general, fetal, and electrophysiology), anesthesiology, intensive care (cardiovascular [CVICU] and neonatal [NICU]), and social work, was held to discuss the case. It was agreed to have a route and timing of delivery based on the usual indications, with follow‐up by maternal fetal medicine every 2 weeks until 36 WGA, then weekly thereafter. Serial ultrasound assessment to monitor fetal growth and a repeated fetal echocardiogram was scheduled at 30 WGA.

For labor and delivery, the plan was arranged as follows:

- Care team huddle to ensure all team members were in agreement with the management plan.
- Collect cord blood sample for genetic testing for CPVT (RyR2).
- Newborn to be monitored in the NICU for risk of arrhythmia and hypoglycemia. Mother opted for breastfeeding to provide beta‐blockade to the newborn, understanding this may be suboptimal.
- Early epidural anesthesia and prophylactic dexmedetomidine were recommended for sympathetic suppression.
- Continuous cardiovascular monitoring.
- Avoidance of sympathomimetics (i.e., terbutaline, epinephrine, and methergine) for the usual labor indications; phenylephrine was the recommended vasopressor if needed.
- Electrolytes goal: magnesium >2 mg/dl, ionized calcium >4.5 mg/dl, and potassium >3.5 mg/dl. This was to prevent confounding causes of ventricular ectopy.
- Esmolol infusion to be initiated depending on the last dose of metoprolol and ectopic burden.
- Flecainide to be continued as scheduled and additional doses could be considered for increased ectopic burden.
- ICD lower ventricular pacing rate adjustment to improve rate regulation (pacing at higher rates).
- If surgical delivery, ICD shock setting should be turned off to avoid possible inappropriate therapy delivery secondary to cauterizer. If so, external defibrillator pads should be placed.
- Maintain open communication with the patient throughout labor to promote the least possible stressful environment.
- Two CVICU nurses to provide collaborative care during labor and delivery.

**Figure 1:**
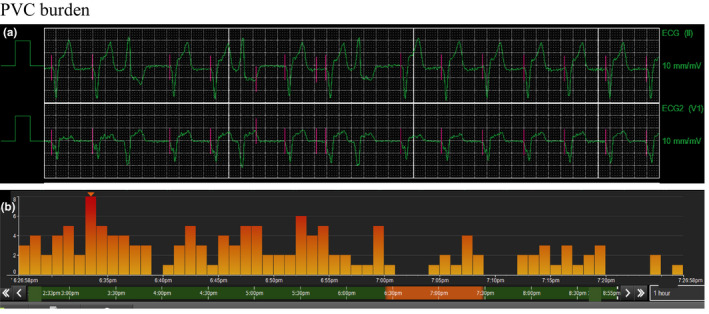
PVC burden. a, Premature ventricular contractions (PVC) with trigeminy pattern. b, Histogram with decreasing PVC burden after increasing esmolol dose

She was admitted at 39 weeks of gestational age for induction of labor. Her ICD interrogation showed two episodes of NSVT in the week prior to admission. During labor, an epidural with bupivacaine was placed, sympathomimetic medication was avoided, and infusions of dexmedetomidine at 0.1 mcg/kg/min and esmolol at 50 mcg/kg/min were started. Given increased polymorphic PVC burden (bigeminy), esmolol was up‐titrated to 150 mcg/kg/min and the ventricular pacing rate was increased to 100 bpm resulting in suppression of ectopy (Figure 1). She delivered a 2608 g female without complications. Esmolol and dexmedetomidine infusions were gradually weaned off within the next 12 hr after metoprolol was restarted. Ventricular pacing was set to baseline configurations 24 hr postpartum. Neonate was observed in the NICU for 48 hr without symptoms. Genetic testing from cord blood was obtained, and results were negative for the maternal mutation.

## 2 DISCUSSION

Catecholaminergic polymorphic ventricular tachycardia is a rare inherited rhythm disorder with an estimated prevalence of about 0.1 per 1,000 people. It could be associated with autosomal dominant transmission (RyR2 gene mutation, CPVT 1) or autosomal recessive transmission (CASQ2 gene mutation, CPVT 2) (Priori, Wilde, & Horie, 2013). This channelopathy is caused by a mutation that provokes excessive intramyocardial calcium release in the setting of a catecholamine surge, and presents with ventricular tachycardia/fibrillation during exercise or emotional stress that can variably manifest from palpitations to sudden death. Pregnancy induces several hormonal, physiologic, and autonomic changes, including increased cardiovascular response to circulating catecholamines (Barron, Mujais, Zinaman, Bravo, & Lindheimer, 1986), and the peripartum is a stage that carries a significant amount of emotional and physical stress.

There is very limited literature regarding the management of patients with CPVT during the peripartum period. Our literature search found only four case reports describing their experience with labor and delivery in patients with polymorphic VT (Ahmed & Phillips, 2016; Burrows, Fox, Biblo, & Roth, 2013; Friday, Moak, Fries, & Iqbal, 2015; Gogle & Kemp, 2018). Given that this is a rare clinical scenario, and there are a lack of guidelines for its management, approaches have varied within all cases reported, including a clinical dilemma about the need to treat or not to treat an asymptomatic patient (Kotschet, Hunter, Kroushev, & Wallace, 2017).

Although there are conflicting data about the amount of catecholaminergic surge in vaginal versus cesarean delivery, research has shown that maternal levels of catecholamines are higher during vaginal delivery compared with cesarean section (Irestedt, Lagercrantz, Hjemdahl, Hagnevik, & Belfrage, 1982); however, the combined pregnancy and postpartum arrhythmic risk in CPVT patients may not be elevated compared with the nonpregnant period (Cheung, Lieve, & Roston, 2019; Roston, van der Werf, & Cheung, 2020). It is known that epidural anesthesia during labor reduces maternal catecholamine levels, probably by eliminating the psychological and physical stress associated with painful uterine contractions (Shnider et al., 1983), reason why our management included early application of this therapy.

Our case presented with a previous history of uncomplicated vaginal delivery that occurred before her cardiac arrest. Although not hemodynamically significant, there was an increase in the frequency of NSVT toward the end of the pregnancy and this was more noticeable during the labor, despite early epidural anesthesia and sympathetic suppression. This increment in her ectopic burden improved after beta‐blockade increase and adjustment of the ventricular pacing rate.

## 3 CONCLUSION

Defining a clear multidisciplinary plan, including anticipated escalation of care for possible complications, helped provide an effective approach to treat this unusual situation on a rare condition and created a level of familiarity among the care providers that certainly contributed to deliver a safer and less stressful experience for both, patient and providers.

## CONFLICT OF INTEREST

The authors declare that there are no relevant or material financial interests that relate to disclose.

## AUTHOR CONTRIBUTION

All authors participated in writing and editing the manuscript.

## ETHICS

The Institutional Review Board of the University of Minnesota waived the need for ethics approval and the need to obtain consent for the collection, analysis, and publication of the retrospectively obtained and anonymized data for this non‐interventional study.
